# Permutation transformations of tensors with an application

**DOI:** 10.1186/s40064-016-3720-1

**Published:** 2016-11-28

**Authors:** Yao-Tang Li, Zheng-Bo Li, Qi-Long Liu, Qiong Liu

**Affiliations:** School of Mathematics and Statistics, Yunnan University, Kunming, 650091 People’s Republic of China

**Keywords:** Permutation transformation, Structure tensor, Weakly irreducible tensor, Canonical form, 15A69, 12E05, 12E10

## Abstract

The permutation transformation of tensors is introduced and its basic properties are discussed. The invariance under permutation transformations is studied for some important structure tensors such as symmetric tensors, positive definite (positive semidefinite) tensors, *Z*-tensors, *M*-tensors, Hankel tensors, *P*-tensors, *B*-tensors and *H*-tensors. Finally, as an application of permutation transformations of tensors, the canonical form theorem of tensors is given. The theorem shows that some problems of higher dimension tensors can be translated into the corresponding problems of lower dimension weakly irreducible tensors so as to handle easily.

## Background

The study of tensors with their various applications has attracted extensive attention and interest, since the work of Qi (2005) and Lim (2005). Lately, the research topic on structure tensors has also attracted much attention, such as symmetric tensors (Qi 2005), $P(P_0)$-tensors (Song and Qi 2014), $B(B_0)$-tensors (Song and Qi 2014), *Z*-tensors (Zhang et al. 2014), (strong) *M*-tensors (Zhang et al. 2014), *H*-tensors (Li et al. 2014) and so on. In the researches on tensors with its application, the reducibility and higher dimension of tensors are two important factors to cause difficulties. Therefore, it is interesting that how to translate problems of higher dimension reducible tensors into the corresponding problems of lower dimension irreducible tensors.

As we all know, the permutation transformation of matrices plays a very important role in linear algebra and matrix theory. Some problems of higher dimension reducible matrices can be translated into the corresponding problems of lower dimension irreducible matrices by using the permutation transformation of matrices. Inspired by this, we introduce permutation transformations of tensors, and discuss its basic properties and and their applications in this paper.

In the next section, we will introduce the permutation transformation of tensors and give its expression. In third section, we will discuss basic properties of permutation transformations of tensors. In fourth section, we will discuss the invariance under permutation transformations for some important structure tensors such as symmetric tensors, positive definite tensors, *M*-tensors, Hankel tensors, *P*-tensors, *B*-tensors, *H*-tensors and so on. In fifth section, we will give the canonical form theorem of tensors and a numerical example which shows that some problems of higher dimension tensors can be translated into the corresponding problems of lower dimension weakly irreducible tensors by using permutation transformations. Finally, we draw some conclusions in the last section.

## Permutation transformations of tensors and its expression

For a positive integer $n$, let $[n]=\{1,2,\ldots ,n\}$. An order $m$ tensor $\mathcal {A}=(a_{i_1\ldots i_m})\in \mathbb {C}^{n_1\times n_2\times \cdots \times n_m}$ is a multidimensional array with $n_1n_2\ldots n_m$ entries, where $i_j\in [n_j],~j\in [m]$. Especially, an order $m$ dimension $n$ tensor $\mathcal {A}=(a_{i_1\ldots i_m})$ over the complex field $\mathbb {C}$ (real field $\mathbb {R}$) consists of $n^m$ complex (real) entries:

$$a_{i_1\ldots i_m}\in \mathbb {C} ~(\mathbb {R}),$$

where $i_j\in [n]$ for $j\in [m]$ (Chang et al. 2008; De Lathauwer et al. 2000; Liu et al. 2010; Ng et al. 2009; Zhang and Golub 2001). It is obvious that a matrix is an order 2 tensor. We shall denote the set of all complex (real) order $m$ dimension $n$ tensors by $\mathbb {C}^{[m,n]}$ ($\mathbb {R}^{[m,n]}$, respectively).

### **Definition 1**

Let $\mathcal {A}=(a_{i_1\ldots i_m})\in \mathbb {C}^{[m,n]},\mathcal {B}=(b_{i_1\ldots i_m})\in \mathbb {C}^{[m,n]}$, and $k\in \mathbb {C}$. Define

(i) $$\mathcal {A}+\mathcal {B}=(a_{i_1\ldots i_m}+b_{i_1\ldots i_m}).$$
(ii) $$k\mathcal {A}=(ka_{i_1\ldots i_m}).$$

### *Remark 1*

Obviously, both $\mathbb {C}^{[m,n]}$ and $\mathbb {R}^{[m,n]}$ are linear spaces about the addition and the multiplication in Definition 1.

### **Definition 2**

(Qi 2005) A tensor $\mathcal {A}=(a_{i_1\ldots i_m})\in \mathbb {R}^{[m,n]}$ is called a symmetric tensor if its entries $a_{i_1\ldots i_m}$ are invariant under any permutation of their indices.

Denote the set of all real order $m$ dimension $n$ symmetric tensors by $\mathbb {S}^{[m,n]}$. Furthermore, $\mathbb {S}^{[m,n]}$ is a linear subspace of $\mathbb {R}^{[m,n]}$. An order $m$ dimension $n$ tensor is called the unit tensor (Yang and Yang 2010), denoted by $\mathcal {I}$, if its entries are $\delta _{i_1\ldots i_m}$ for $i_1,\ldots ,i_m \in [n]$, where

$$\delta _{i_1\ldots i_m}=\left\{ \begin{array}{ll} 1, & \quad if\; i_1=\cdots =i_m, \\ 0, &\quad otherwise. \end{array} \right.$$

Let $\mathcal {A}=(a_{i_1\ldots i_m})\in \mathbb {R}^{[m,n]}$ and $x\in \mathbb {R}^n$. Then $\mathcal {A}x^m$ is a homogeneous polynomial of degree $m$, defined by

$$\mathcal {A}x^{m}=\sum \limits _{i_1,\ldots ,i_m\in [n]}a_{i_1\ldots i_m}x_{i_1}\ldots x_{i_m}.$$

A tensor $\mathcal {A}\in \mathbb {R}^{[m,n]}$ is called positive semidefinite (Song and Qi 2014) if for any vector $x \in \mathbb {R}^n,\mathcal {A}x^m\ge 0$, and it is called positive definite if for any nonzero vector $x \in \mathbb {R}^n,\mathcal {A}x^m>0$.

Now, we give the definition of permutation transformation of tensors.

### **Definition 3**

Let $\mathcal {A}=(a_{i_1\ldots i_m})\in \mathbb {C}^{[m,n]}$ and $\pi$ be a permutation on $[n]$, we define $P_\pi :\mathbb {C}^{[m,n]} \rightarrow \mathbb {C}^{[m,n]}$ by

$$P_\pi (\mathcal {A})=(a_{\pi (i_1)\ldots \pi (i_m)}).$$

$P_\pi$ is called as a permutation transformation on $\mathbb {C}^{[m,n]}$, and is simply called as a permutation transformation. $P_\pi (\mathcal {A})$ is called as the image of $\mathcal {A}$ under $P_\pi$.

### *Remark 2*

$P_\pi$ is called as a permutation transformation on $\mathbb {R}^{[m,n]}$ if $\mathbb {C}^{[m,n]}$ is replaced by $\mathbb {R}^{[m,n]}$ in Definition 3.

### **Definition 4**

Let $\mathcal {A}=(a_{i_1\ldots i_m})\in \mathbb {C}^{[m,n]}$ and $\pi ^{-1}$ be the inverse permutation of $\pi$ on $[n]$, we define $P_\pi ^{-1}:\mathbb {C}^{[m,n]}\rightarrow \mathbb {C}^{[m,n]}$ by

$$P_\pi ^{-1}(\mathcal {A})=P_{\pi ^{-1}}(\mathcal {A}).$$

$P_\pi ^{-1}$ is called as the inverse permutation transformation of $P_\pi$ on $\mathbb {C}^{[m,n]}$, and is simply called as the inverse permutation transformation.

For further discussing property of the permutation transformation of tensors, we introduce the following general product of two $n$-dimensional tensors defined in Shao (2013). For the sake of simplicity, we sometime use the following “condensed notation” for the subscripts of the tensor. For example, we will write $a_{i_1i_2\ldots i_m}$ as $a_{i_1\alpha }$, where $\alpha =i_2\ldots i_m\in [n]^{m-1}$and $[n]^{m-1}$ is $m-1$ dimensional array whose every element varies from 1 to $n$.

### **Definition 5**

(Shao 2013) Let $\mathcal {A}$ and $\mathcal {B}$ be order $m\ge 2$ and order $k\ge 1$, dimension $n$ tensors, respectively. Define the product $\mathcal {A}\cdot \mathcal {B}$ (sometimes simplified as $\mathcal {A}\mathcal {B}$) to be the following tensor $\mathcal {C}$ of order $(m-1)(k-1)+1$ and dimension $n$,

$$c_{i\alpha _1\ldots \alpha _{m-1}}=\sum \limits _{i_2,\ldots ,i_m\in [n]} a_{ii_2\ldots i_m}b_{i_2\alpha _1}\ldots b_{i_m\alpha _{m-1}},$$

where $$i\in [n],\alpha _1,\ldots ,\alpha _{m-1}\in [n]^{k-1}$$.

Especially, when $P=(p_{ij})$ and $Q=(q_{ij})$ are both matrices, we have the following formula (Shao 2013):

$$(P\mathcal {A}Q)_{i_1\ldots i_m}=\sum \limits _{j_1,\ldots ,j_m\in [n]} a_{j_1\ldots j_m}p_{i_1j_1}q_{j_2i_2}\ldots q_{j_mi_m}.$$

### **Definition 6**

(Shao 2013) Let $\mathcal {A}$, $\mathcal {B}\in \mathbb {C}^{[m,n]}$. If there exists a permutation $\pi$ on the set $[n]$, and the corresponding permutation matrix $P=(P_{ij})$ (where $P_{ij}=1 \Longleftrightarrow j=\pi (i)$; $P_{ij}=0$, otherwise.) such that $\mathcal {B}=P \mathcal {A}P^T$, then we say that $\mathcal {A}$ and $\mathcal {B}$ are permutational similar.

### *Remark 3*

(Shao 2013) If $\mathcal {A}$, $\mathcal {B}$ are permutational similar, then

(i) $$b_{i_1\ldots i_m}=a_{\pi (i_1)\ldots \pi (i_m)}$$,
(ii) $$P \mathcal {I}P^T=\mathcal {I}$$.

### **Definition 7**

(Shao 2013) Let $\mathcal {A},\mathcal {B}\in \mathbb {C}^{[m,n]}$. Suppose that there exist two matrices $P$ and $Q$ of dimension $n$ with $P\mathcal {I}Q=\mathcal {I}$ such that $\mathcal {B}=P\mathcal {A}Q$, then we say that the two tensors $\mathcal {A}$ and $\mathcal {B}$ are similar.

Now, we present the relationship between the permutation transformation of tensors and permutational similar.

### **Theorem 1**

*Let* $\mathcal {A},\mathcal {B}\in \mathbb {C}^{[m,n]}$. *Then* $\mathcal {A}$ *and* $\mathcal {B}$ *are permutational similar if and only if there exists a permutation* $\pi$ *on* $[n]$ *such that* $\mathcal {B}=P_\pi (\mathcal {A})$.

### *Proof*

Assume that $\mathcal {A}$ and $\mathcal {B}$ are permutational similar. By Remark 3, there exists a permutation $\pi$ on $[n]$ such that $b_{i_1\ldots i_m}=a_{\pi (i_1)\ldots \pi (i_m)}$. Define a permutation transformation $P_\pi : \mathbb {C}^{[m,n]}\rightarrow \mathbb {C}^{[m,n]}$ by

$$P_\pi (\mathcal {A})=(a_{\pi (i_1)\ldots \pi (i_m)}),$$

which implies $$\mathcal {B}=P_\pi (\mathcal {A})$$.

On the other hand, assume that there exists a permutation transformation $P_\pi$ such that $\mathcal {B}=P_\pi (\mathcal {A})$, then

$$b_{i_1\ldots i_m}=a_{\pi (i_1)\ldots \pi (i_m)}.$$

Let permutation matrix $P=P_\pi$ $(P_{ij}=1 \Longleftrightarrow j=\pi (i);~P_{ij}=0,~otherwise.)$ corresponding to $\pi$. Then

$$\begin{aligned} (P\mathcal {A}P^T)_{i_1\ldots i_m}&=\sum \limits _{j_1,\ldots ,j_m\in [n]}a_{j_1\ldots j_m}P_{i_1j_1}(P^T)_{j_2i_2}\ldots (P^T)_{j_mi_m}\\&=a_{\pi (i_1)\ldots \pi (i_m)}.\end{aligned}$$

Hence, $\mathcal {B}=P\mathcal {A}P^T$. Thus, $\mathcal {A}$ and $\mathcal {B}$ are permutational similar. $\square$

By Theorem 1 and Remark 3, we have the following expression theorem of permutational transformation of tensors.

### **Theorem 2**

*Let* $\mathcal {A}\in \mathbb {C}^{[m,n]}$, *and* $\pi$ *be a permutation on* $[n]$. *Then*

$$P_\pi (\mathcal {A})=P\mathcal {A}P^T,$$

*where* $$P_{ij}=1 \Longleftrightarrow j=\pi (i)$$; $$P_{ij}=0$$, *otherwise.*

## Basic properties of permutation transformations

Now, we discuss basic properties of permutation transformation of tensors. Firstly, we present some definitions and a lemma, which are needed in the subsequent analysis. For an *n*-dimensional vector $x=(x_1,x_2,\ldots ,x_n)$, real or complex, we define the *n*-dimensional vector:

$$\mathcal {A}x^{m-1} := \left( \sum \limits _{i_2,\ldots ,i_m\in [n]} a_{ii_2\ldots i_m}x_{i_2}\ldots x_{i_m}\right) _{1\le i \le n},$$

and the *n*-dimensional vector:

$$x^{[m-1]} := \left( x_i^{m-1}\right) _{1\le i \le n}.$$

The following two definitions were first introduced and studied by Lim (2005) and Qi (2005).

### **Definition 8**

(Lim 2005; Qi 2005) Let $\mathcal {A}\in \mathbb {R}^{[m,n]}$. A pair $(\lambda ,x)\in \mathbb {C}\times (\mathbb {C}^{n}\setminus \{0\})$ is called an eigenvalue-eigenvector (or simply eigenpair) of $\mathcal {A}$ if they satisfy the equation

1 $$\mathcal {A}x^{m-1}=\lambda x^{[m-1]}.$$

We call $(\lambda ,x)$ an H-eigenpair if they are both real.

The set of all eigenvalues of $\mathcal {A}$ is denoted by $\sigma (\mathcal {A})$ and it is called the spectral of $\mathcal {A}$. Let $\rho (\mathcal {A})=\max \{|\lambda |:\lambda \in \sigma (\mathcal {A})\}$. It is called the spectral radius of $\mathcal {A}$.

### **Definition 9**

(Lim 2005; Qi 2005) Let $\mathcal {A}\in \mathbb {R}^{[m,n]}$. A pair $(\lambda ,x)\in \mathbb {C}\times (\mathbb {C}^{n}\setminus \{0\})$ is called an E-eigenvalue and E-eigenvector (or simply E-eigenpair) of $\mathcal {A}$ if they satisfy the equation

2 $$\left\{ \begin{array}{ll} \mathcal {A}x^{m-1}=\lambda x, \\ x^{T}x=1. \end{array}\right.$$

We call $(\lambda ,x)$ an Z-eigenpair if they are both real.

### **Definition 10**

(Zhang et al. 2014) A tensor $\mathcal {A}=(a_{i_1\ldots i_m})\in \mathbb {R}^{[m,n]}$ is called strictly diagonally dominant if

$$\mid a_{ii\ldots i}\mid > \mathop {\mathop {\sum }\limits _{i_2,\ldots , i_m\in [n],}}\limits _{\delta _{ii_2\ldots i_m=0}} \mid a_{ii_2\ldots i_m}\mid ,\quad \forall i\in [n].$$

### **Definition 11**

(Bu et al. 2014) A tensor $\mathcal {A}\in \mathbb {C}^{n_1\times \cdots \times n_k}$ is said to have rank-one if there exist nonzero $a_i\in \mathbb {C}^{n_i}(i=1,\ldots ,k)$ such that $\mathcal {A}= a_1\otimes a_2\otimes \cdots \otimes a_k$, where $a_1\otimes a_2\otimes \cdots \otimes a_k$ is the segre outer product of $a_1\in \mathbb {C}^{n_1},\ldots ,a_k\in \mathbb {C}^{n_k}$ with entries $a_{i_1\ldots i_k}= (a_1)_{i_1}\ldots (a_k)_{i_k}$. The rank of a tensor $\mathcal {A}$, denoted by $rank(\mathcal {A})$, is defined to be the smallest $r$ such that $\mathcal {A}$ can be written as a sum of $r$ rank-one tensors. Especially, if $\mathcal {A}=0$, then $rank(\mathcal {A})=0$.

### **Lemma 1**

(Qi 2005) *Assume that* $\mathcal {A}\in \mathbb {R}^{[m,n]}$ *is an even-order symmetric tensor. The following conclusions hold for* $\mathcal {A}$,

(i) ${\mathcal {A}}$ *always has H-eigenvalues*. $\mathcal {A}$ *is positive definite (positive semidefinite) if and* *only if all of its H-eigenvalues are positive* (*nonnegative*).
(ii) $\mathcal {A}$ *always has Z-eigenvalues*. $\mathcal {A}$ *is positive definite* (*positive semidefinite*) *if and only if all of its Z-eigenvalues are positive* (*nonnegative*).

Next, we present some basic properties of permutation transformation of tensor as follows.

### **Theorem 3**

*Let* $\mathcal {A}$, $\mathcal {B}\in \mathbb {C}^{[m,n]}~(\mathbb {R}^{[m,n]})$, *and* $\pi$ *be a permutation on* $[n]$. *Then*

(i) $$P_\pi (\mathcal {A}+\mathcal {B})=P_\pi (\mathcal {A})+P_\pi (\mathcal {B})$$.
(ii) $$P_\pi (k\mathcal {A})=kP_\pi (\mathcal {A})$$, *where* $$k\in \mathbb {C}~(\mathbb {R})$$.
(iii) $$P_\pi (\mathcal {I})=\mathcal {I}$$.
(iv) $$P_\pi ^{-1}(P_\pi (\mathcal {A}))=\mathcal {A}$$.
(v) $$P_\pi (P_\pi ^{-1}(\mathcal {A}))=\mathcal {A}$$.
(vi) $$\sigma (P_\pi (\mathcal {A}))=\sigma (\mathcal {A})$$.
(vii) $$\rho (P_\pi (\mathcal {A}))=\rho (\mathcal {A})$$.
(viii) $$rank(P_\pi (\mathcal {A}))=rank(\mathcal {A})$$.
(ix) *If* $\mathcal {A}\in \mathbb {S}^{[m,n]}$, *then* $P_\pi (\mathcal {A})\in \mathbb {S}^{[m,n]}$.
(x) If $\mathcal {A}$ *is a strictly diagonally* *dominant tensor, then* $P_\pi (\mathcal {A})$ *is also a strictly diagonally dominant tensor*.
(xi) *If m is even and* $\mathcal {A}\in \mathbb {S}^{[m,n]}$ *is positive definite* (*positive semidefinite*) tensor, then $P_\pi (\mathcal {A})\in \mathbb {S}^{[m,n]}$ and is *also a positive definite* (*positive semidefinite*) *tensor*.

### *Proof*

(i) $$P_\pi (\mathcal {A}+\mathcal {B})_{i_1\ldots i_m}=a_{\pi (i_1)\ldots \pi (i_m)}+b_{\pi (i_1)\ldots \pi (i_m)}=\big (P_\pi (\mathcal {A})+P_\pi (\mathcal {B})\big )_{i_1\ldots i_m}.$$
(ii) $$P_\pi (k\mathcal {A})_{i_1\ldots i_m}=ka_{\pi (i_1)\ldots \pi (i_m)}=kP_\pi (\mathcal {A})_{i_1\ldots i_m}.$$
(iii) Since $P_\pi (\mathcal {I})_{i_1\ldots i_m}=\mathcal {I}_{\pi (i_1) \ldots \pi (i_m)}$ and $\delta _{\pi (i_1)\ldots \pi (i_m)}=1$ if and only if $\delta _{i_1\ldots i_m}=1$, then $P_\pi (\mathcal {I})=\mathcal {I}$.
  From the Definition 3, it is easy to obtain (iv) and (v) are hold.
(vi) By Theorem 1, $P_\pi (\mathcal {A})$ and $\mathcal {A}$ are permutational similar. By Theorem 2.1 in Shao (2013),
  $$\phi _{P_\pi (\mathcal {A})}(\lambda )=\phi _{\mathcal {A}}(\lambda ),$$
  where $\phi _{\mathcal {A}}(\lambda )$ is the characteristic polynomial of the tensor $\mathcal {A}$. In Qi (2005), Qi has proved that a number $\lambda \in \mathbb {C}$ is an eigenvalue of $\mathcal {A}$ if and only if it is a root of $\phi _{\mathcal {A}}(\lambda )$. Hence, similar tensors have the same eigenvalues. Then
  $$\sigma (\mathcal {A})=\sigma (P_\pi (\mathcal {A})).$$
(vii) It is easy to be got from the results of (vi).
(viii) Let $rank(\mathcal {A})=r$, and $P_\pi (\mathcal {A})=(a_{\pi (i_1)\ldots \pi (i_m)})$, where $\pi$ is a permutation on $[n]$. **Case 1**. If $r=1$, then there exists $a_i\in \mathbb {C}^{n_i}~(i\in [m],a_i\ne 0)$ such that
  $$\mathcal {A}=a_1\otimes a_2\otimes \cdots \otimes a_m$$
  which implies,
  $$a_{i_1\ldots i_m}=(a_1)_{i_1}(a_2)_{i_2}\ldots (a_m)_{i_m}.$$
  Therefore,
  $$\begin{aligned} a_{\pi (i_1)\pi (i_2)\ldots \pi (i_m)}&=(a_1)_{\pi (i_1)}(a_2)_{\pi (i_2)}\ldots (a_m)_{\pi (i_m)}\\&=(P_\pi (a_1))_{i_1}(P_\pi (a_2))_{i_2}\ldots (P_\pi (a_m))_{i_m}.\end{aligned}$$
  Hence, $P_\pi (\mathcal {A})={P_\pi (a_1)}\otimes {P_\pi (a_2)}\otimes \cdots \otimes {P_\pi (a_m)}$. Since $P_\pi (a_i)\in \mathbb {C}^{n_i}$ and $P_\pi (a_i)\ne 0$, then $rank(P_\pi (\mathcal {A}))=1$.**Case 2**. If $r>1$, then $\mathcal {A}$ can be written at least as a sum of $r$ rank-one tensors. Let $\mathcal {A}=\sum \nolimits _{i\in [r]}\mathcal {A}_i$, where $\mathcal {A}_i\in \mathbb {C}^{[m,n]},rank(\mathcal {A}_i)=1,i\in [r]$. Then,
  $$P_\pi (\mathcal {A})=\sum \limits _{i\in [r]}P_\pi (\mathcal {A}_i).$$
  By the results of Case 1, we have
  $$rank(P_\pi (\mathcal {A}_i))=1,i\in [r].$$
  Hence, $rank(P_\pi (\mathcal {A}))\le r$. Next, we will prove that $rank(P_\pi (\mathcal {A}))< r$ is impossible. Suppose that $rank(P_\pi (\mathcal {A}))=r'<r$. Then $P_\pi (\mathcal {A})$ can be written at least as a sum of $r'$ rank-one tensors as follows
  $$P_\pi (\mathcal {A})=\sum \limits _{i\in [r']}\mathcal {D}_i,\quad where\quad \mathcal {D}_i\in \mathbb {C}^{[m,n]},\quad rank(\mathcal {D}_i)=1,i\in [r'],$$
  and $\pi ^{-1}$ be the inverse transformation of $\pi$ on $[n]$. Then from $(iv)$,
  $$\mathcal {A}=P_\pi ^{-1}(P_\pi (\mathcal {A}))= \sum \limits _{i\in [r']}P_{\pi ^{-1}}(\mathcal {D}_i).$$
  From case 1, $rank(P_\pi ^{-1}(\mathcal {D}_i))=1$, so $rank(\mathcal {A})\le r'< r$. It’s a contradiction. Therefore, $rank(P_\pi (\mathcal {A}))=r$.
(ix) It is easy to be proved from the definition of symmetric tensors.
(x) Suppose that $\mathcal {A}$ is a strictly diagonally dominant tensor, then
  $$\begin{aligned}\mid P_\pi (\mathcal {A})_{ii\ldots i}\mid&=\mid \mathcal {A}_{ \pi (i)\pi (i)\ldots \pi (i)}\mid \\&> \mathop {\mathop {\sum }\limits _{i_2,\ldots , i_m\in [n],}}\limits _{{\delta _{\pi (i)i_2\ldots i_m=0}}} \mid a_{\pi (i)i_2\ldots i_m}\mid \\&= \mathop {\mathop {\sum }\limits _{i_2,\ldots , i_m\in [n],}}\limits _{\delta _{ii_2\ldots i_m=0}} \mid P_\pi (\mathcal {A})_{ii_2\ldots i_m}\mid,~\forall i\in [n]. \end{aligned}$$
  Thus, $P_\pi (\mathcal {A})$ is also a strictly diagonally dominant tensor.
(xi) Suppose that $\mathcal {A}$ is positive definite (semidefinite), then all H-eigenvalues of $\mathcal {A}$ are positive (nonnegative). By (vi) and (ix) of Theorem 3, $P_\pi (\mathcal {A})$ is an even-order symmetric tensor, and $\sigma (P_\pi (\mathcal {A}))=\sigma (\mathcal {A})$. From the results above, we know that all H-eigenvalues of $P_\pi (\mathcal {A})$ are positive (nonnegative). Then, by Lemma 1, $P_\pi (\mathcal {A})$ is positive definite (positive semidefinite).

$$\square$$

### *Remark 4*

By the results of (i) and (ii) of Theorem 3, we know that a permutation transformation of tensor is a linear transformation on $\mathbb {C}^{[m,n]}~(\mathbb {R}^{[m,n]})$.

## Permutation transformation on some structure tensors

It is universally acknowledged that some structure tensors with good properties have been well studied, such as nonnegative tensor, symmetric tensor, positive definite (positive semidefinite) tensor, $Z$-tensor, (strong) $M$-tensor, Hankel tensor, $P(P_0)$-tensor, $B(B_0)$-tensor and $H$-tensor. In this section, we discuss the invariance under permutation transformations for some important structure tensors.

### **Definition 12**

(Chang et al. 2008) A tensor $\mathcal {A}=(a_{i_1\ldots i_m})\in \mathbb {R}^{[m,n]}$ is called a nonnegative tensor, denoted by $\mathcal {A}\ge 0$, if each entry is nonnegative.

### **Definition 13**

(Zhang et al. 2014) We call a tensor $\mathcal {A}$ as an $Z$-tensor, if all of its off-diagonal entries are non-positive, which is equivalent to write $\mathcal {A}=s\mathcal {I}-\mathcal {B}$, where $s>0$ and $\mathcal {B}$ is a nonnegative tensor.

From Definition 12, we easily get the following two lemmas.

### **Lemma 2**

*Let* $\mathcal {A}\in \mathbb {R}^{[m,n]}$ *be a nonnegative tensor,* $\pi$ *be a permutation on* $[n]$. *Then* $P_\pi (\mathcal {A})$ *is also a nonnegative tensor*.

### **Lemma 3**

*Let* $\mathcal {A}\in \mathbb {R}^{[m,n]}$ *be an* $Z$-*tensor, and* $\pi$ *be a permutation on* $[n]$. *Then* $P_\pi (\mathcal {A})$ *is also an* $Z$-*tensor.*

### **Definition 14**

(Zhang et al. 2014) We call an $Z$-tensor $\mathcal {A}=s\mathcal {I}-\mathcal {B}(\mathcal {B}\ge 0)$ as an $M$-tensor if $s\ge \rho (\mathcal {B})$; we call it as a strong $M$-tensor if $s>\rho (\mathcal {B})$, where $\rho (\mathcal {B})$ is the spectral radius of $\mathcal {B}$.

### **Theorem 4**

*Let* $\mathcal {A}\in \mathbb {R}^{[m,n]}$ *be an* (*strong*) $M$-*tensor, and* $\pi$ *be a permutation on* $[n]$. *Then* $P_\pi (\mathcal {A})$ *is also an (strong)* $M$-*tensor*.

### *Proof*

Suppose that $\mathcal {A}\in \mathbb {R}^{[m,n]}$ is an (strong) $M$-tensor, then there exist a nonnegative tensor $\mathcal {B}$ and a real number $s\ge \rho (\mathcal {B})(s>\rho (\mathcal {B}))$ such that

$$\mathcal {A}=s\mathcal {I}-\mathcal {B}.$$

From Remark 3,

$$P_\pi (\mathcal {A})=s\mathcal {I}-P_{\pi }(\mathcal {B}).$$

By Lemma 3, $P_\pi (\mathcal {A})$ is an $Z$-tensor. It follows from (vii) of Theorem 3 that $\rho (P_{\pi }(\mathcal {B}))=\rho (\mathcal {B})$, which implies

$$s\ge \rho (P_\pi (\mathcal {B}))~~(s>\rho (P_\pi (\mathcal {B}))).$$

Therefore, $P_{\pi }(\mathcal {A})$ is an (strong) $M$-tensor. $\square$

### **Definition 15**

(Song and Qi 2014) Let $\mathcal {A}=(a_{i_1i_2\ldots i_m})\in \mathbb {R}^{[m,n]}$. We say that $\mathcal {A}$ is

(i) a $P_0$-tensor if for any nonzero vector $x\in \mathbb {R}^n$, there exists $i\in [n]$ such that $x_i\ne 0$ and $x_i(\mathcal {A}x^{m-1})_i\ge 0$;
(ii) a $P$-tensor if for any nonzero vector $x\in \mathbb {R}^n$, $\max \nolimits _{i\in [n]}x_i(\mathcal {A}x^{m-1})_i>0$.

### **Lemma 4**

(Song and Qi 2014) *A symmetric tensor is a* $P(P_0)$-*tensor if and only if it is positive (semi)definite.*

### **Theorem 5**

*Let* $\mathcal {A}\in \mathbb {S}^{[m,n]}$ *be an even-order* $P(P_0)$-*tensor, and* $\pi$ *be a permutation on* $[n]$. *Then* $P_\pi (\mathcal {A})$ *is also an even-order symmetric* $P(P_0)$-*tensor*.

### *Proof*

Suppose that $\mathcal {A}$ is an even-order symmetric $P(P_0)$-tensor. According to Lemma 4, $\mathcal {A}$ is positive (semi)definite. It follows from (ix) of Theorem 3 that $P_\pi (\mathcal {A})$ is positive (semi)definite. Hence, by Lemma 4, $P_\pi (\mathcal {A})$ is a $P(P_0)$-tensor. $\square$

### **Definition 16**

(Song and Qi 2014) Let $\mathcal {A}=(a_{i_1i_2\ldots i_m})\in \mathbb {R}^{[m,n]}$. We say that $\mathcal {A}$ is a $B$-tensor, if for all $i\in [n]$,

$$\sum \limits _{i_2,\ldots ,i_m\in [n]}a_{ii_2\ldots i_m}>0,$$

and

$$\frac{1}{n^{m-1}}\left( \sum \limits _{i_2,\ldots ,i_m\in [n]}a_{ii_2\ldots i_m}\right) >a_{ij_2\ldots j_m},$$

for all $(j_2,\ldots ,j_m)\ne (i,\ldots ,i)$. We say that $\mathcal {A}$ is a $B_0$-tensor, if for all $i\in [n]$,

$$\sum \limits _{i_2,\ldots ,i_m\in [n]}a_{ii_2\ldots i_m}\ge 0,$$

and

$$\frac{1}{n^{m-1}}\left( \sum \limits _{i_2,\ldots ,i_m\in [n]}a_{ii_2\ldots i_m}\right) \ge a_{ij_2\ldots j_m},$$

for all $$(j_2,\ldots ,j_m)\ne (i,\ldots ,i)$$.

### **Theorem 6**

*Let* $\mathcal {A}\in \mathbb {R}^{[m,n]}$ *be a* $B(B_0)$-*tensor, and* $\pi$ *be a permutation on* $[n]$. *Then* $P_\pi (\mathcal {A})$ *is also a* $B(B_0)$-*tensor*.

### *Proof*

Suppose that $\mathcal {A}=(a_{i_1i_2\ldots i_m})$ is a $B$-tensor, then for all $i\in [n]$,

$$\sum \limits _{i_2,\ldots ,i_m\in [n]}a_{ii_2\ldots i_m}>0,$$

and

$$\frac{1}{n^{m-1}}\left( \sum \limits _{i_2,\ldots ,i_m\in [n]}a_{ii_2\ldots i_m}\right) >a_{ij_2\ldots j_m},$$

for all $$(j_2,\ldots ,j_m)\ne (i,\ldots ,i)$$.

Let $$P_\pi (\mathcal {A})=(b_{i_1i_2\ldots i_m})$$. Then

$$\begin{aligned} \sum \limits _{i_2,\ldots ,i_m\in [n]}b_{ii_2\ldots i_m}&=\sum \limits _{i_2,\ldots ,i_m\in [n]}a_{\pi (i)\pi (i_2)\ldots \pi (i_m)}\\ &=\sum \limits _{\pi (i_2),\ldots ,\pi (i_m)\in [n]}a_{\pi (i)\pi (i_2)\ldots \pi (i_m)}>0, \end{aligned}$$

and

$$\begin{aligned} \frac{1}{n^{m-1}}\left( \sum \limits _{i_2,\ldots ,i_m\in [n]}b_{ii_2\ldots i_m}\right)&=\frac{1}{n^{m-1}}\left( \sum \limits _{i_2,\ldots ,i_m\in [n]}a_{\pi (i)\pi (i_2)\ldots \pi (i_m)}\right) \\&=\frac{1}{n^{m-1}}\left( \sum \limits _{\pi (i_2),\ldots ,\pi (i_m)\in [n]}a_{\pi (i)\pi (i_2)\ldots \pi (i_m)}\right) \\&>a_{\pi (i)\pi (j_2)\ldots \pi (j_m)}=b_{ij_2\ldots j_m}. \end{aligned}$$

Hence, $P_\pi (\mathcal {A})$ is a $B$-tensor. Similarly, we can prove that the case of $B_0$-tensor. $\square$

### **Definition 17**

(Li et al. 2014) A tensor $\mathcal {A}=(a_{i_1i_2\ldots i_m})\in \mathbb {C}^{[m,n]}$ is called an $H$-tensor if there is an entrywise positive vector $x=(x_1,x_2,\ldots ,x_n)^T\in \mathbb {R}^n$ such that for all $i\in [n]$,

3 $$\mid a_{i\ldots i}\mid x_i^{m-1}> \mathop {\mathop {\sum }\limits _{i_2,\ldots ,i_m\in [n],}}\limits _{\delta _{ii_2\ldots i_m}=0} \mid a_{ii_2\ldots i_m}\mid x_{i_2}\ldots x_{i_m}.$$

### **Theorem 7**

*Let* $\mathcal {A}\in \mathbb {C}^{[m,n]}$ *be an* $H$-*tensor, and* $\pi$ *be a permutation on* $[n]$. *Then* $P_\pi (\mathcal {A})$ *is also an* $H$-*tensor*.

### *Proof*

Suppose that $\mathcal {A}=(a_{i_1i_2\ldots i_m})$ is an $H$-tensor. Then there is an entrywise positive vector $x=(x_1,x_2,\ldots ,x_n)^T\in \mathbb {R}^n$ such that for all $i\in [n]$, the Inequality 3 holds. Let $P_\pi (\mathcal {A})=(b_{i_1i_2\ldots i_m})$, where $b_{i_1i_2\ldots i_m}=a_{\pi (i_1)\pi (i_2)\ldots \pi (i_m)}$. Hence, for all $i\in [n]$,

$$\begin{aligned} \mid b_{i\ldots i}\mid x_i^{m-1}= & {} \mid a_{\pi (i)\ldots \pi (i)}\mid x_i^{m-1}\\> & {} \mathop {\mathop {\sum }\limits _{i_2,\ldots , i_m\in [n],}}\limits _{\delta _{\pi (i)i_2\ldots i_m}=0} \mid a_{\pi (i)i_2\ldots i_m}\mid x_{i_2}\ldots x_{i_m}\\= & {} \mathop {\mathop {\sum }\limits _{i_2,\ldots , i_m\in [n],}}\limits _{\delta _{ii_2\ldots i_m}=0} \mid b_{ii_2\ldots i_m}\mid x_{i_2}\ldots x_{i_m}\\= & {} \mathop {\mathop {\sum }\limits _{i_2,\ldots ,i_m\in [n],}}\limits _{\delta _{ii_2\ldots i_m}=0} \mid b_{ii_2\ldots i_m}\mid x_{i_2}\ldots x_{i_m}. \end{aligned}$$

Thus, $P_\pi (\mathcal {A})$ is an $H$-tensor. $\square$

## Canonical form of tensors

In this section, as an application of the permutation transformation of tensors, we would introduce some results which show that the canonical form theorem for matrices could be generalized to tensors. For the convenience discussion, we starts with the following definitions and lemmas.

Let $\mathcal {A}\in \mathbb {R}^{[m,n]}$ be a nonnegative tensor. The directed graph $G(\mathcal {A})$ (Chang et al. 2013; Friedland et al. 2013) associated to $\mathcal {A}$ is the directed graph with vertices $1,2,\ldots , n$ and an edge from $i$ to $j$ if and only if $a_{ii_2\ldots i_m}\ne 0$ for some $i_l=j,l=2,3,\ldots ,m.$

### **Definition 18**

(Chang et al. 2013; Friedland et al. 2013; Hu et al. 2014) A nonnegative tensor $\mathcal {A}\in \mathbb {R}^{[m,n]}$ is called weakly irreducible if the associate directed graph $G(\mathcal {A})$ is strongly connected. A tensor $\mathcal {A}$ is said to be weakly irreducible if $|\mathcal {A}|$ is weakly irreducible, where $|\mathcal {A}|$ denote the tensor whose $(i_1,\ldots ,i_m)$-th entry is defined as $|a_{i_1\ldots i_m}|$.

### **Definition 19**

(Shao et al. 2013) Let $\mathcal {A}=(a_{i_1\ldots i_m})\in \mathbb {R}^{[m,n]}$. If there exists some integer $k$ with $1\le k\le n-1$ such that

$$a_{i_1i_2\ldots i_m}=0,$$

for all $i_1\le k$ and at least one of $\{i_2,\ldots ,i_m\}$ is greater than $k$, then $\mathcal {A}$ is called a $k$-lower triangular block tensor, or simply a lower triangular block tensor.

### **Definition 20**

Let $\mathcal {A}\in \mathbb {R}^{[m,n]}$ and $\alpha \subseteq [n]$. A principal subtensor $\mathcal {A}[\alpha ]$ of the tensor $\mathcal {A}$ is defined as an order $m$ dimensional $|\alpha |$ tensor with entries

$$\mathcal {A}[\alpha ]=(a_{i_1\ldots i_m}),\quad i_1,\ldots ,i_m\in \alpha .$$

### **Definition 21**

(Shao et al. 2013) Let $\mathcal {A}\in \mathbb {R}^{[m,n]}$, and $n_1,\ldots ,n_r$ be positive integers with $n_1+\cdots +n_r=n~(r\ge 1)$. Let

$$\begin{aligned} I_1 &= {} \{1,2,\ldots ,n_1\},\\ I_i & = {} \left\{ \left( \sum \limits _{j\in [i-1]}{n_j}\right) +1,\ldots ,\sum \limits _{j\in [i]}{n_j}\right\} \subseteq [n]~(i\in [r]\setminus \{1\}), \end{aligned}$$

and write $\mathcal {A}[I_i]=\mathcal {A}_i$. Suppose that for each $1\le i\le r-1$, the subtensor $\mathcal {A}[I_i\cup \cdots \cup I_r]$ is a $n_i$-lower triangular block tensor, then $\mathcal {A}$ is called a $(n_1,\ldots ,n_r)$-lower triangular block tensor with the diagonal blocks $\mathcal {A}_1,\ldots ,\mathcal {A}_r$.

### **Lemma 5**

(Shao et al. 2013) *Let* $\mathcal {A}\in \mathbb {R}^{[m,n]}$ *and* $m\ge 2$. *Then there exists positive integers* $r\ge 1$ *and* $n_1,\ldots ,n_r$ *with* $n_1+\cdots +n_r=n (r\ge 1)$ *such that* $\mathcal {A}$ *is permutational similar to some* $(n_1+\cdots +n_r)$-*lower triangular block tensor, where all the diagonal blocks* $\mathcal {A}_1,\ldots ,\mathcal {A}_r$ *are weakly irreducible.*

### **Lemma 6**

(Shao et al. 2013) *Let* $r\ge 2$ *and* $n_1,\ldots ,n_r$ *be positive integers with* $n_1+\cdots +n_r=n$. *Let* $\mathcal {A}$ *be a* $(n_1+\cdots +n_r)$-*lower triangular block tensor with the diagonal blocks* $\mathcal {A}_1,\ldots ,\mathcal {A}_r$. *Then we have:*

$$Det(\mathcal {A})=\prod \limits _{i\in [r]}(Det\mathcal {A}_i)^{(m-1)^{n-n_i}},$$

*and thus*

$$\phi _{\mathcal {A}}(\lambda )=\prod \limits _{i\in [r]}(\phi _{\mathcal {A}_i}(\lambda ))^{(m-1)^{n-n_i}},$$

*where* $\phi _{\mathcal {A}}(\lambda )$ *is the characteristic polynomial of the tensor* $\mathcal {A}$.

From Theorem 1, Lemmas 5 and 6, we can easily obtain the following theorems.

### **Theorem 8**

*Let* $\mathcal {A}\in \mathbb {R}^{[m,n]}$, *then there exists a permutation* $\pi$ *on* $[n]$ *such that* $P_\pi (\mathcal {A})$ *is a* $(n_1+\cdots +n_r)$-*lower triangular block tensor, where all the diagonal blocks* $\mathcal {A}_1,\ldots ,\mathcal {A}_r$ *are weakly irreducible*.

### *Remark 5*

Theorem 8 could be called the “canonical form” theorem and the $(n_1+\cdots +n_r)$-lower triangular block tensor is called the “canonical form” of $\mathcal {A}$.

### **Theorem 9**

*Let* $\mathcal {A}\in \mathbb {R}^{[m,n]}$ *and* $\overline{\mathcal {A}}$ *be the “canonical form” of* $\mathcal {A}$. *Then*

$$\sigma (\mathcal {A})=\sigma (\overline{\mathcal {A}})= \bigcup \limits _{i\in [r]}\sigma (\mathcal {A}_i).$$

### *Proof*

From Theorem 8 and (vi) of Theorem 3, we have

$$\sigma (\mathcal {A})=\sigma (\overline{\mathcal {A}}).$$

From Lemma 6, we have

$$\sigma (\overline{\mathcal {A}})=\bigcup \limits _{i\in [r]}\sigma (\mathcal {A}_i).$$

Therefore, the conclusion holds. $\square$

Canonical form of tensors plays an important role in decomposition of symmetric tensors as a sum of component rank-one tensors, which has numerous applications in electrical engineering and higher order statistics (Brachat et al. 2009; Kolda and Bader 2009; Robeva 2016), such Independent Component Analysis (Comon 1992). Given a symmetric tensor $\mathcal {A}\in \mathbb {R}^{[m,n]}$, the aim is to decompose it as

4 $$\mathcal {A}=\sum \limits _{i\in [k]}\lambda _i v_i^{\bigotimes m},$$

where $v_1,\ldots , v_k\in \mathbb {R}^{n}$ and $\lambda _1,\ldots ,\lambda _k\in \mathbb {R}$. Comon et al. (2008) showed that the decomposition of the form (4) exists. Furthermore, in Brachat et al. (2009) an algorithm for decomposition a symmetric tensor into a sum of component rank-one tensors was presented. However, for a tensor with large order or dimension the algorithm may result in large computing quantity. According to Theorem 8, and taking into account that $\mathcal {A}$ is symmetric, there exists a permutation $\pi$ on $[n]$ such that $P_\pi (\mathcal {A})$ is a diagonal block tensor, where all the diagonal blocks $\mathcal {A}_1\in \mathbb {R}^{[m,n_1]},\ldots ,\mathcal {A}_r\in \mathbb {R}^{[m,n_r]}(\sum \nolimits _{i\in [r]}n_i=n)$ are weakly irreducible. Therefore, we can use algorithm in Brachat et al. (2009) decomposition of lower dimensional symmetric tensor $\mathcal {A}_j,j\in [r]$ as follows

$$\mathcal {A}_j=\sum \limits _{i\in [k_j]}\lambda _{ji} v_{ji}^{\bigotimes m},\quad j\in [r],v_{ji}\in \mathbb {R}^{n_j}.$$

Therefore,

$$P_\pi (\mathcal {A})=\sum \limits _{j\in [r]}\sum \limits _{i\in [k_j]}\lambda _{ji} \omega _{ji}^{\bigotimes m},$$

where

$$\omega _{ji}=(\overbrace{0,\ldots ,0}^{n_1 \text {times}},\ldots \overbrace{0,\ldots ,0}^{n_{j-1}\text {times}},(v_{ji})^{T},\overbrace{0,\ldots ,0}^{n_{j+1}\text {times}}, \ldots ,\overbrace{0,\ldots ,0}^{n_r\text {times}})^{T}\in \mathbb {R}^{n},j\in [r],i\in [k_j],$$

thus, we obtain decomposition of the tensor $\mathcal {A}$ as follows.

$$\mathcal {A}=\sum \limits _{j\in [r]}\sum \limits _{i\in [k_j]}\lambda _{ji} \left( P^{-1}_\pi (\omega _{ji})\right) ^{\bigotimes m}.$$

We shall employ an example to illustrate this interesting property.

### *Example 1*

Let $\mathcal {A}=(a_{i_1i_2i_3i_4})\in \mathbb {R}^{[4,4]}$ be a symmetric tensor defined by

$$\begin{aligned}&a_{1111}=3,a_{2222}=15,a_{3333}=18,a_{1333}=a_{3133}=a_{3313}=a_{3331}=6,\\&a_{1133}=a_{1313}=a_{1331}=a_{3113}=a_{3131}=a_{3311}=6,\\&a_{2224}=a_{2242}=a_{2422}=a_{4222}=9, a_{2444}=a_{4244}=a_{4424}=a_{4442}=3,\\&a_{2244}=a_{2424}=a_{2442}=a_{4224}=a_{4242}=a_{4422}=3,\text {other}, a_{i_1i_2i_3 i_4}=0. \end{aligned}$$

By Theorem 8, we can obtain the canonical form of $\mathcal {A}$ as follows. Let $\pi$ be a permutation on $\{1,2,3,4\}$, where $\pi (1)=1,\pi (2)=3,\pi (3)=2,\pi (4)=4$. Therefore, $P_\pi (\mathcal {A})=(b_{i_1i_2i_3i_4})\in \mathbb {R}^{[4,4]}$ reads as follows

$$\begin{aligned}&b_{1111}=3,b_{2222}=18,b_{3333}=15,b_{1222}=b_{2122}=b_{2212}=b_{2221}=6,\\&b_{1122}=b_{1212}=b_{1221}=b_{2112}=b_{2121}=b_{2211}=6,\\&b_{3334}=b_{3343}=b_{3433}=b_{4333}=9, b_{3444}=b_{4344}=b_{4434}=b_{4443}=3,\\&b_{3344}=b_{3434}=b_{3443}=b_{4334}=b_{4343}=b_{4433}=3,\text {other}, b_{i_1i_2i_3 i_4}=0. \end{aligned}$$

It is easy to obtain that $P_\pi (\mathcal {A})$ is a diagonal block tensor with the diagonal blocks $P_\pi (\mathcal {A})[I_j],j\in \{1,2\}$, where $I_1=\{1,2\},I_2=\{3,4\}$. By algorithm 1 in Brachat et al. (2009), we obtain the rank-one decomposition of the diagonal block tensors $P_\pi (\mathcal {A})[I_j],j\in \{1,2\}$ as follows

$$\begin{aligned} P_\pi (\mathcal {A})[I_1] &= {} v_{11}^{\bigotimes 4}+2v_{12}^{\bigotimes 4},\\ P_\pi (\mathcal {A})[I_2] &= {} v_{21}^{\bigotimes 4}-v_{22}^{\bigotimes 4}, \end{aligned}$$

where

$$v_{11}=(1,2)^{T},\quad v_{12}=(1,-1)^{T},\quad v_{21}=(2,1)^{T},\quad v_{22}=(1,-1)^{T}.$$

Therefore

$$P_\pi (\mathcal {A})=\omega _{11}^{\bigotimes 4}+2\omega _{12}^{\bigotimes 4}+\omega _{21}^{\bigotimes 4}-\omega _{22}^{\bigotimes 4},$$

where

$$\begin{aligned} \omega _{11} &=(v_{11}^{T},0,0)^{T}=(1,2,0,0)^{T}, \quad \omega _{12}=(v_{12}^{T},0,0)^{T}=(1,-1,0,0)^{T},\\ \omega _{21} & =(0,0, v_{21}^{T})^{T}=(0,0,2,1)^{T},\quad \omega _{22}=(0,0, v_{22}^{T})^{T}=(0,0,1,-1)^{T}. \end{aligned}$$

which implies the rank-one decomposition of tensor $\mathcal {A}$ given by

$$\mathcal {A}=(P_\pi ^{-1}(\omega _{11}))^{\bigotimes 4}+2(P_\pi ^{-1}(\omega _{12}))^{\bigotimes 4}+(P_\pi ^{-1}(\omega _{21}))^{\bigotimes 4}-(P_\pi ^{-1}(\omega _{22}))^{\bigotimes 4},$$

where,

$$\begin{aligned} P_\pi ^{-1}(\omega _{11}) & =(1,0,2,0)^{T},\quad P_\pi ^{-1}(\omega _{12})=(1,0,-1,0)^{T},\\ P_\pi ^{-1}(\omega _{21})& =(0,2,0,1)^{T},\quad P_\pi ^{-1}(\omega _{22})=(0,1,0,-1)^{T}. \end{aligned}$$

## Conclusions

In this paper, the definition of permutation transformation of tensors is introduced, and its properties are studied. As applications of permutation transformations, we give the canonical form theorem of tensors and a numerical example which show that some problems of higher dimension tensors can be translated into the corresponding problems of lower dimension weakly irreducible tensors by using the permutation transformation. There are many problems unsolved for permutation transformations of tensors and their applications. Hence, this paper is only a starting point for studying permutation transformations of tensors.
